# Skin Disease Recognition Method Based on Image Color and Texture Features

**DOI:** 10.1155/2018/8145713

**Published:** 2018-08-26

**Authors:** Li-sheng Wei, Quan Gan, Tao Ji

**Affiliations:** School of Electrical and Engineer, Anhui Polytechnic University, Wuhu 241000, China

## Abstract

Skin diseases have a serious impact on people's life and health. Current research proposes an efficient approach to identify singular type of skin diseases. It is necessary to develop automatic methods in order to increase the accuracy of diagnosis for multitype skin diseases. In this paper, three type skin diseases such as herpes, dermatitis, and psoriasis skin disease could be identified by a new recognition method. Initially, skin images were preprocessed to remove noise and irrelevant background by filtering and transformation. Then the method of grey-level co-occurrence matrix (GLCM) was introduced to segment images of skin disease. The texture and color features of different skin disease images could be obtained accurately. Finally, by using the support vector machine (SVM) classification method, three types of skin diseases were identified. The experimental results demonstrate the effectiveness and feasibility of the proposed method.

## 1. Introduction

Composed of epidermis, dermis, and subcutaneous tissues, skin is the largest organ of human body, containing blood vessels, lymphatic vessels, nerves, and muscles, which can perspire, perceive the external temperature, and protect the body. Covering the entire body, the skin can protect multiple tissues and organs in the body from external invasions including artificial skin damage, chemical damage, adventitious viruses, and individuals' immune system. Besides, skin can also avoid the loss of lipids together with water within epidermis and dermis so that skin barrier function can be stabilized [1]. In spite of defense and barrier function, skin is not indestructible in that skin tends to be constantly influenced by a variety of external and genetic factors. Currently, there are three main types of skin diseases appearing in human body, including viral skin diseases, fungal skin diseases, and allergic skin disease. Despite the fact that these types of skin diseases can be cured at present, these diseases indeed have brought trouble to patients' life. Nowadays, the majority of conclusions on the patients' existing symptoms are drawn mainly based on doctors' years of experience or their own subjective judgments, which may lead to misjudgments and consequently delay the treatment of these. Therefore, it is of great theoretical significance and practical value to study how to extract symptoms of diverse skin diseases on the basis of modern science and technology. Under this circumstance, effective and accurate identification of the types of skin diseases can be achieved to prescribe treatment according to patients' symptoms [2].

Over the past few years, the image processing technique has achieved rapid development in medicine. Some equipment based on digital image technology has also been widely applied to people's everyday life, for instance, computed tomography (CT), digital subtraction angiography (DSA), and magnetic resonance imaging (MRI). Deeper research on this direction has been carried out by scholars all over the world. For example, the skin disease varicella was detected by Oyola and Arroyo [3] through image processing technique's color transformation, equalization as well as edge detection, and the image of varicella was eventually collected and classified through Hough transform. The final empirical results demonstrated that a better diagnosis was received in terms of detection on varicella, and preliminary test was also conducted on varicella and herpes zoster on that basis. Chung and Sapiro [4] put forward a method to detect the image of skin lesions based on partial differential equation (PDE), with which a contour model of skin lesions was extracted on the basis of its morphological filtering through PDE. The final empirical results demonstrated that skin diseases could be accurately identified through this algorithm. Yu et al. [5] made a diagnosis on herpes simplex, varicella, and herpes zoster through reflectance confocal microscopy (RCM). The final empirical results demonstrated that specificity could be extracted from all the three different types of herpes. Zhong et al. [6] diagnosed psoriasis vulgaris through three-dimensional computed tomography (CT) imageological technique of skin. The final empirical results demonstrated that diagnosis on psoriasis vulgaris of Munro's microabscess was highly sensitive and specific. Sumithra et al. [7] proposed a novel approach for automatic segmentation and classification of skin lesions by using SVM and k-nearest neighbor (k-NN) classifier. Yasir et al. [8] proposed a detection system, which could be used in computers or mobile phones, based on computer vision techniques. Arivazhagan et al. [9] proposed an automated system based on texture analysis for recognizing human skin diseases by independent component analysis of skin color image. The minimum distance classifier was used to classify the type of human skin diseases. Niu et al. [10] carried out an experiment on the color image of skin erythema through HSV color space and fuzzy C-means clustering algorithm. The final empirical results demonstrated that the time and accuracy of final segmentation could be improved through this method of dimensionality reduction. Liu and Guo [11] diagnosed and identified skin tumors, vascular dermatosis, and psoriasis through computed tomography (CT) based on the principle of optical confocal, which has been widely applied. Luo et al. [12] conducted diagnosis and identification on vitiligo through an independently developed analytic system of skin digital images. Both scientific and objective, the quantitative evaluation method was worth studying. Lu et al. [13] classified smooth pixels by employing two-dimensional digital image segmentation and resizing combined with Markov random field (MRF), which demonstrated a reliable segmentation. The final results illustrated that the diagnosis and identification of psoriasis can be well solved. Jaleel et al. [14] researched the skin cancer diagnosing methodology by using back propagation neural (BPN) network classifier. Salimi et al. [15] presented the pattern recognition method to classify the skin disease. Ganeshkumar and Vasanthi [16] researched the melanoma disease detection technique by using preprocessing and edge detection. Kolkur et al. [17] proposed a new human skin detection algorithm to improve the recognition of skin pixels, such as RGB (red, green, and blue), HSV (hue, saturation, and value), and YCbCr (luminance and chrominance) color models. Gindhi et al. [18] and Kotian and Deepa [19] studied the problem of skin disease automated diagnosis system based on the techniques of image border identification and feature data mining by matlab software. Kumar and Singh [20] established the relationship of skin cancer images across different types of neural network. Then, medical images were collected into this skin cancer classification system for training and testing based on the matlab image processing toolbox.

The above literature has made significant achievements on the identification of skin diseases. However, the proposed methods mainly aim at the identification of one type of skin disease, which makes them difficult to apply to the precise identification of multitype skins. Therefore, in this paper, a method based on vertical image segmentation, GLCM, and SVM is proposed to identify three various types of skin diseases, namely, herpes, paederus dermatitis, and psoriasis. Firstly, the sample images of three skin diseases need to be preprocessed. Secondly, the vertical image is segmented and made corresponding geometric transformation. Based on this, three types of skin diseases' features are extracted, and their correlated parameters of feature texture and pixels of lesion areas are collected through image segmentation. Finally, the symptoms of herpes, paederus dermatitis, and psoriasis are identified by utilizing the support vector machine (SVM) method in order to improve identification accuracy.

The remaining structures of this paper are as follows. The Preliminaries section presents the identification method of skin diseases based on GLCM and SVM. The feature extraction and disease recognition are presented in detail. Experimental results, comparisons, and discussions are provided in the Experiments and Analysis section, and finally conclusions are given in the Conclusions section.

## 2. Preliminaries

**Figure 1:**
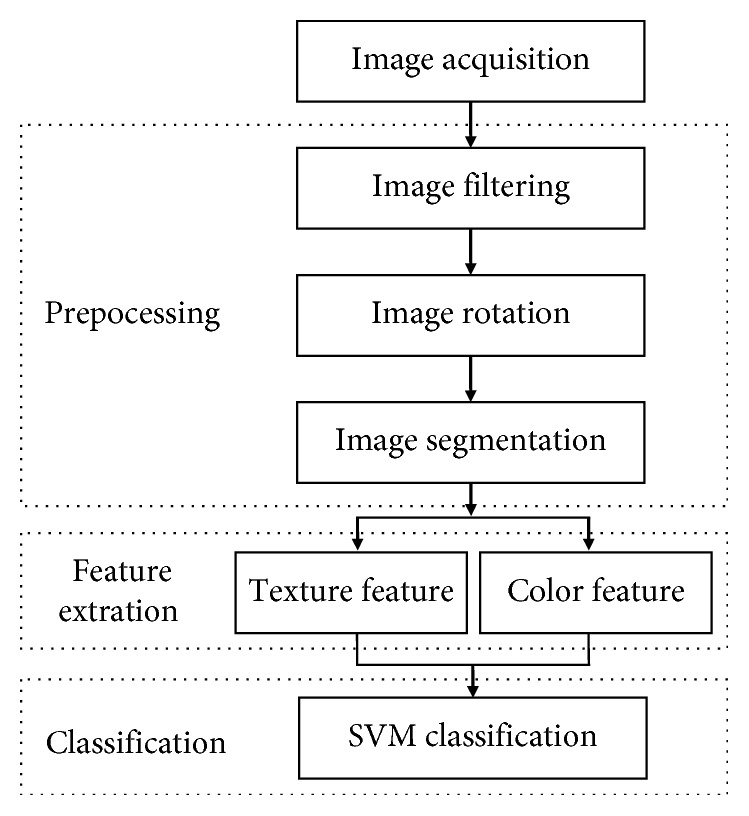
The identification process of skin diseases.

The main steps of proposed methodology to skin disease recognition are shown in Figure 1. The flow chart contains three phases: (1) processing of the original image; (2) feature extraction; and (3) classification based on SVM. The first stage is image processing. Because image may contain some unwanted noise, it becomes necessary to filter the image to remove the noise. Then, by using image rotation and segmentation, the representation of an image into something that is more meaningful and easier to analyze. The specific areas of skin lesions are precisely divided, and the identification accuracy is improved through vertical image segmentation. The second stage is feature extraction, in which the image texture features and color features of the skin lesions are further extracted. In texture feature extraction, GLCM is used to find the mathematical parameters of like contrast, correlation, entropy, uniformity, and energy. The third stage is to identify the three various types of skin diseases according to obtained features based on SVM.

### 2.1. Image Prepocessing

Three common skin diseases are selected in this paper as the main research objects, which are herpes, paederus dermatitis, and psoriasis, respectively. Due to differences in ways of acquiring the source images of skin epidermis and in resolution and size, image preprocessing on source images are needed for the sake of subsequent vertical image segmentation. The specific processing is as follows.

Firstly, considering that noise constantly exerts a negative impact on acquired samples of skin epidermis's source images, it is necessary to denoise through median filtering for the reduction of the impact on skin segmentation and identification brought by irrelevant background in the images. The median filter is a popular way to remove “salt-and-pepper” noise from an image and at the same time preserve edges and keep useful information. In this paper, the median filter is adopted to preprocess and smoothen the source images. The used formula is as follows:

(1) $$\begin{array}{c} F^{\prime }\left(x_{0},y_{0}\right)={\left[\underset{\left(x,y\right)\in S}{\text{sort}}F\left(x,y\right)\right]}_{\left(N+1\right)/2},\;N\geq 0, \end{array}$$

where *F*′(*x*_0_, *y*_0_) is the median of the gray value of the image; *S* is the neighborhood collection of pixel; (*x*, *y*) is the element of *S*; *F*(*x*, *y*) is the gray value of (*x*, *y*); *N* is expressed as the number of the elements in the set of *S*; sort is expressed as sequencing; and [*g*(•)]_(*N*+1)/2_ is the median of the function *g*(•). The main idea of the median filter algorithm is to run through the signal gray value by gray value, replacing each gray value with the median of neighboring gray values. The pattern of neighbors is called the “window,” which slides, gray value by gray value, over the entire signal. By using median filtering algorithm, the original images are denoised and their qualities are enhanced.

Secondly, denoise images are rotated in order to get the medial axis to segment the images. The denoised skin epidermis's source images are processed via neighborhood sampling with the intention of better obtaining the highlight line through the transformation of Euclidean distance. The denoise image *I* is rotated to a specific angle *θ*, in which the width and height of new image *I*_c_ as shown in Equation (2) via transformation of neighborhood sampling are acquired:

(2) $$\begin{array}{c} {\text{width}}_{\text{new}}=2\times \left(\left|\left(\frac{\text{width}}{2\times \cos\;\theta }\right)\right|+\left|\left(\frac{\text{height}}{2\times \sin\;\theta }\right)\right|\right),\\ {\text{height}}_{\text{new}}=2\times \left(\left|\left(\frac{\text{height}}{2\times \cos\;\theta }\right)\right|+\left|\left(\frac{\text{width}}{2\times \sin\;\theta }\right)\right|\right), \end{array}$$

where width and height each represent width and height of the original image, while width_new_ and height_new_ refer to width and height of the rotated image and *θ* is the angle needing rotating.

**Figure 2:**
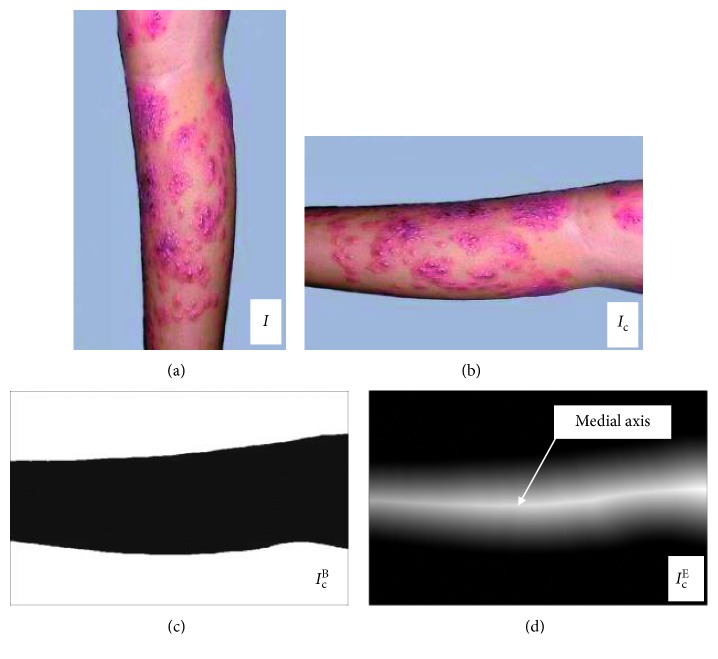
Image of the transformation of Euclidean distance. (a) Filtering image. (b) Rotated image. (c) Binary mask image. (d) European transformation image.

The corresponding coordinates of image *I* and image *I*_c_ is as follows:

(3) $$\begin{array}{c} \left(x_{0}-x_{r1}\right)=\left(x_{1}-x_{r2}\right)\times \cos\;\theta -\left(y_{1}-y_{r2}\right)\times \sin\;\theta ,\\ \left(y_{0}-y_{r1}\right)=\left(x_{1}-x_{r2}\right)\times \sin\;\theta -\left(y_{1}-y_{r2}\right)\times \cos\;\theta , \end{array}$$

where *x*_0_ and *y*_0_ are the original coordinates, while *x*_1_ and *y*_1_ are transformed ones; *x*_*r*1_ and *y*_*r*1_ are the central coordinates of the original image, while *x*_*r*2_ and *y*_*r*2_ are the ones of transformed image. Then, the image of neighborhood sampling transformation is processed via transformation of Euclidean distance which is widely applied to the binary image and is particularly effective for the extraction of skeleton. Accordingly, in this paper, the sample new transformation image *I*_c_ is binarized first to obtain the brightest pixel line, and the transformation of Euclidean distance comes second. The specific process of the transformation of Euclidean distance is as follows: through defining a binary image S under a two-dimensional (2D) *M* × *N*, a background point set *B*(*B* ∈ *S*) is first assumed, and then a foreground point set *F*(*F* ∈ *S*) meeting the premise *B* ∪ *F*=*S* is also assumed. Based on this, *M* is taken as a set in the medial axis transform meeting the premise *M* ∈ *F*. As a result, the shortest distance from a random point *p*_*x*,*y*_ ∈ *M* to *B* can be calculated through Equation (4):

(4) $$\begin{array}{c} d\left(p_{x,y},\;\;b_{m,n}\right)=\sqrt{{\left(x-m\right)}^{2}+{\left(y-n\right)}^{2}}, \end{array}$$

where *d*(•) represents the shortest Euclidean distance; *p*_*x*,*y*_ is a random point in the medial axis transform set; *x* and *y* are the coordinate values of the current point; *b*_*m*,*n*_ is a point belonging to the background point set; and *m* and *n* are the coordinate values of the current point. Binary mask image *I*_c_^B^ is obtained from new image *I*_c_ of neighborhood sampling transformation, and *I*_c_^E^ is eventually acquired via the transformation of Euclidean distance. The brightest pixel line can be obtained from the transformation of via the application of *I*_c_^E^ Euclidean distance, which is medial axis. In Figure 2(b), the image *I*_c_ is rotated to the horizontal coordinate. The binary mask image *I*_c_^B^ is shown in Figure 2(c). And, the Euclidean distance transform image *I*_c_^E^ is presented in Figure 2(d). In order to obtain the vertical segment image, the medial axis is marked out in Figure 2(d).

**Figure 3:**
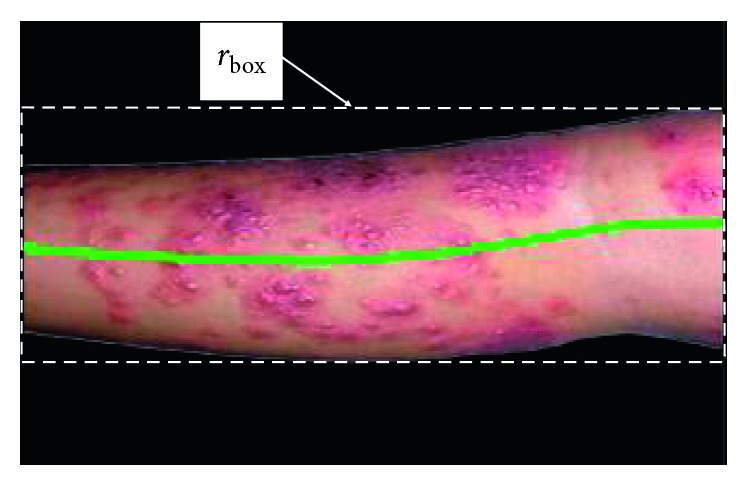
The boundary rectangle of the image and medial axis.

Finally, medial axis of the segmented region is located and divided into ten vertical segments. It is necessary to create a bounding rectangle *r*_box_ on the image *I*_c_ in order to limit the image zone that is dotted with the white line. In Figure 3, it is shown that the medial axis is marked in light green after being extracted from the transformation Euclidean distance.

**Figure 4:**
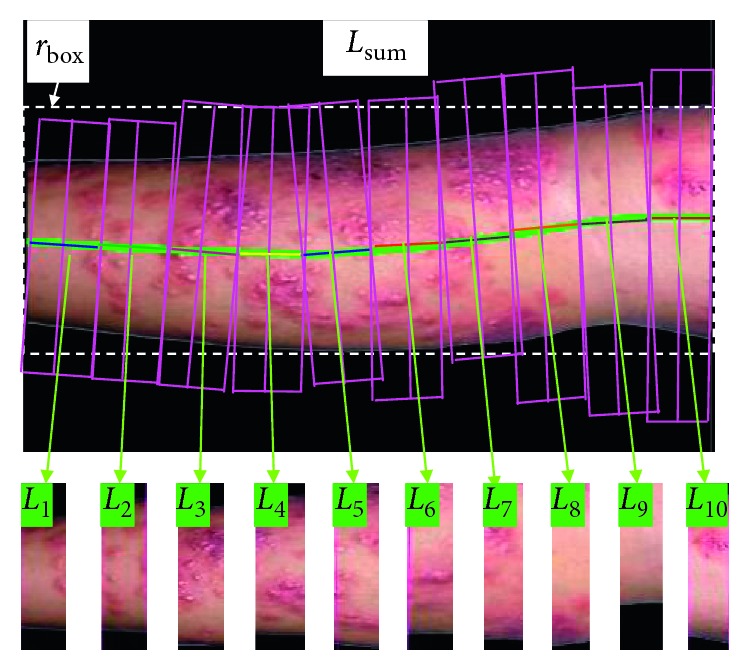
Ten vertical image regions (*L*_1_, *L*_2_,…, *L*_10_).

The perpendicular line for each point on the main axis can be determined. So, the epithelium can be divided into ten vertical image regions, as done in [21]. The correspondingly original medial axis is changed into ten short straight lines, which is shown in Figure 4. After the ten vertical segment images are determined, we will deal with these local images to recognize the images *L*_1_, *L*_2_, …, *L*_10_ of skin disease.

### 2.2. Texture Feature Extraction

**Figure 5:**
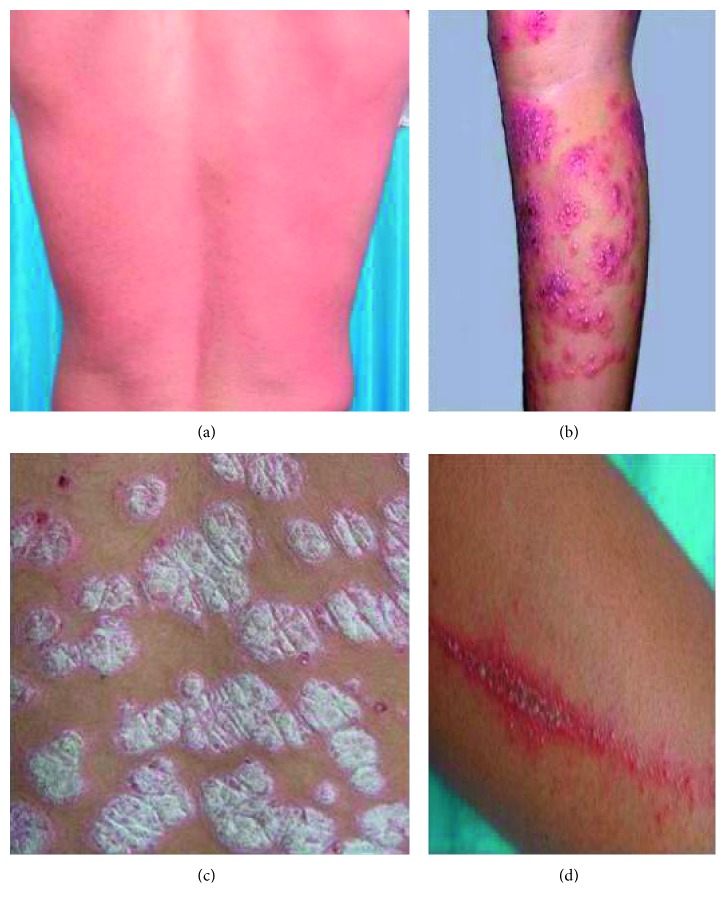
Types of skin diseases. (a) Normal skin. (b) Herpes. (c) Paederus dermatitis. (d) Psoriasis.

Compared with the traditional way, GLCM is an effective tool to analyze the features of texture. The textures of different diseases in the skin epithelial image can be obtained, such as contrast, correlation, entropy, uniformity, and energy. In this paper, three common skin diseases are selected as the main research objects, which are herpes, paederus dermatitis, and psoriasis, respectively. All the pictures as shown in Figure 5 are extracted from photo gallery of sino-medicine website.

#### 2.2.1. Contrast

**Figure 6:**
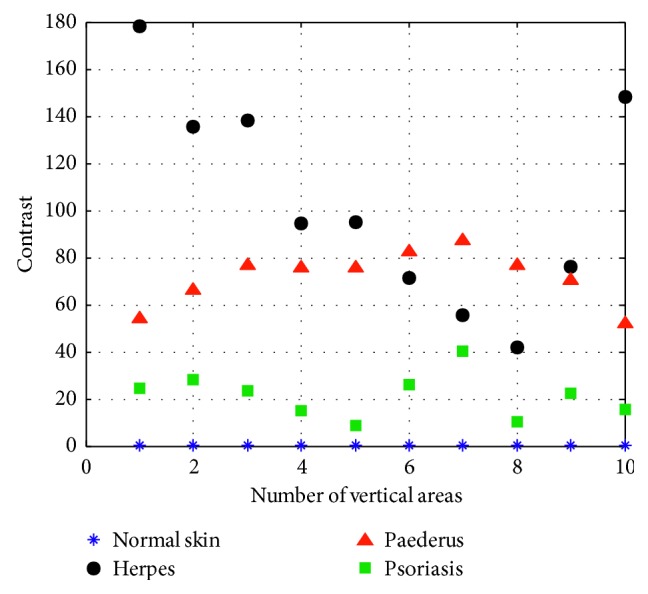
Contrast.

(5) $$\begin{array}{c} A_{1}=\overset{L-1}{\underset{i}{\sum }}\overset{L-1}{\underset{j}{\sum }}{\left(i-j\right)}^{2}G\left(i,j\right), \end{array}$$

where |*i* − *j*| refers to the gray level difference between adjacent pixels; *G*(*i*, *j*) is the distribution probability of gray-level difference between adjacent pixels; and *A*_1_ refers to the contrast, mainly used to describe the degree of depth of the image textile grooves. The higher the contrast value goes, the deeper the grooves, and vice versa. Through experiment, we can get the texture parameter of contrast of normal skin, herpes, paederus, and psoriasis which is depicted in Figure 6.

#### 2.2.2. Correlation

**Figure 7:**
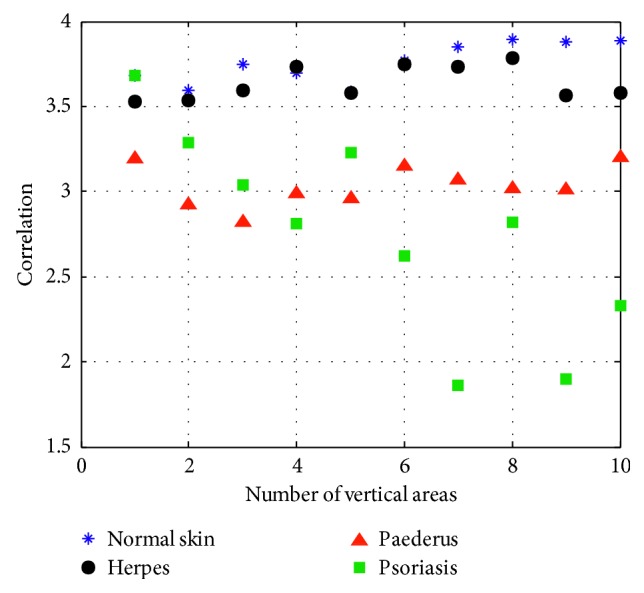
Correlation.

(6) $$\begin{array}{c} A_{2}=\frac{\sum_{i}^{L-1}\sum_{j}^{L-1}\left(i-\bar{x}\right)\left(j-\bar{y}\right)G\left(i,\;\;j\right)}{\sigma_{x}\sigma_{y}}, \end{array}$$

where *A*_2_ refers to correlation, $\bar{x}$ is the mean value of the sum of the elements in each column in the square, *y*_1_ is the mean value of the sum of the elements in each row, *σ*_*x*_ the standard deviation of the sum of the elements in each column, and *σ*_*y*_ the standard deviation of the sum of the elements in each row. Correlation is mainly used to describe the details of relevant elements in each row and column in the process of vertical image segmentation. Then, we have the texture parameter of contrast in Figure 7.

#### 2.2.3. Entropy

**Figure 8:**
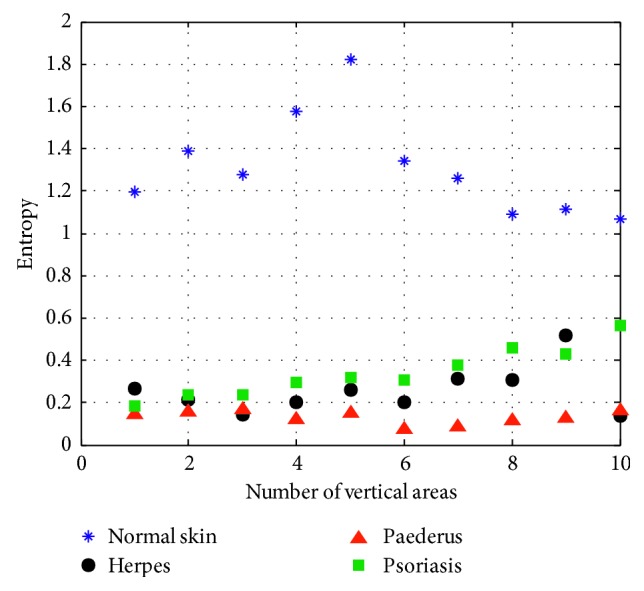
Entropy.

(7) $$\begin{array}{c} A_{3}=-\overset{L-1}{\underset{i}{\sum }}\overset{L-1}{\underset{j}{\sum }}\left[G\left(i,j\right)\cdot \log\;\;G\left(i,j\right)\right], \end{array}$$

where *A*_3_ refers to entropy, which measures the quantity of information within the image and is changed with different textures. As *A*_3_ increases, the textures of speck would be arranged sparsely, and vice versa. When the entropy becomes zero, there is no texture (Figure 8).

#### 2.2.4. Uniformity

**Figure 9:**
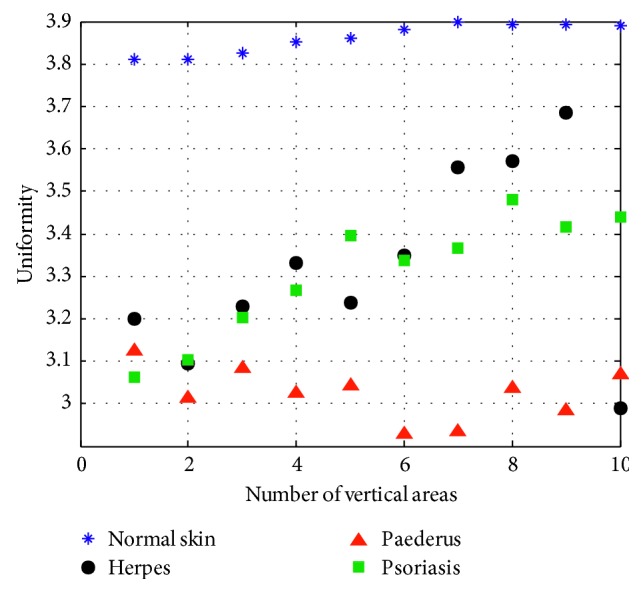
Uniformity.

(8) $$\begin{array}{c} A_{4}=\overset{L-1}{\underset{i}{\sum }}\overset{L-1}{\underset{j}{\sum }}\frac{1}{{\left(i-j\right)}^{2}+1}G\left(i,j\right), \end{array}$$

where *A*_4_ stands for the moment of inertia that is used for the description of the roughness of image texture. The larger the value of *A*_4_, the rougher the texture of endemic area will be, and vice versa (Figure 9).

#### 2.2.5. Energy

**Figure 10:**
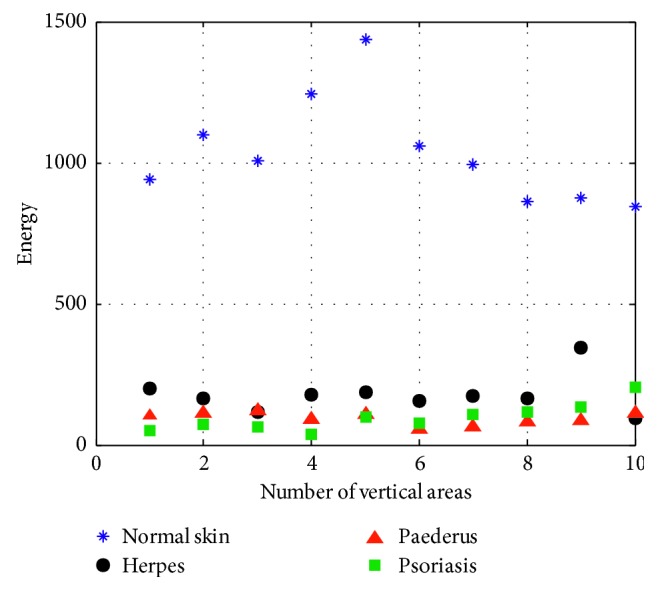
Energy.

(9) $$\begin{array}{c} A_{5}=\overset{L-1}{\underset{i}{\sum }}\overset{L-1}{\underset{j}{\sum }}G^{2}\left(i,j\right), \end{array}$$

where *A*_5_, the energy value mainly applied to describe the thickness of texture, is the quadratic sum of elements of gray-level co-occurrence matrix in the horizontal and vertical directions. We can get the texture parameter of energy as it is depicted, respectively, in Figure 10.

From the above five figures, it can be seen that there are discrepancies in the discrete spots presented by different lesions.  Table 1 shows the texture features of different skin disease for the convenience of comparison.

### 2.3. Color Feature Extraction

**Figure 11:**
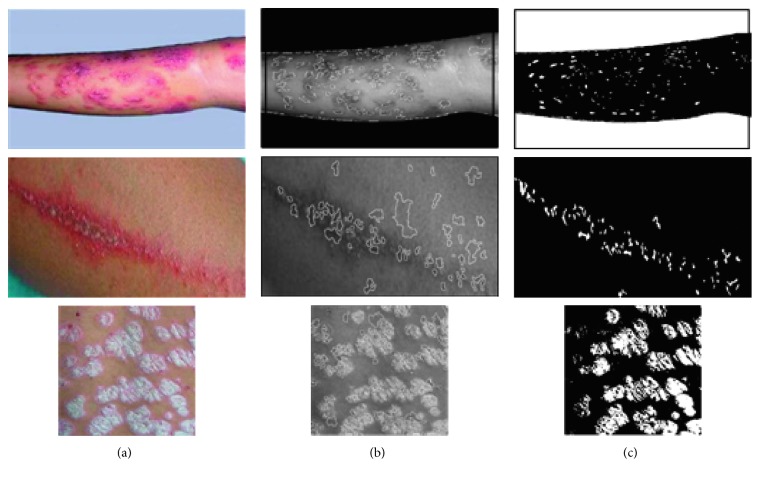
Image segmentation. (a) Original pictures. (b) Marker-controlled watershed segmentation. (c) Mark control + clustering segmentation.

In order to improve identification and treatment, the disease area of different skin diseases can be extracted on the basis of identifying the texture features so that the area of herpes, paederus, and psoriasis can be calculated, correspondingly. Given the fact that most skin diseases appear in the shape of a circle, the watershed algorithm can be applied for image segmentation. In this paper, in order to avoid oversegmentation, marker-controlled watershed, combined with clustering, was performed to segment the image. That is to say, to segment the image by means of watershed algorithm that goes well with a proper threshold, cut each image into relatively small zones, as *S*_*i*_,  *i*=1,  2, …, *W*. In the meantime, areas that are mistakenly divided need to be highlighted. Then, it is required to initialize the feature data of these areas such as *S*_*i*_,  *i*=1,  2, …, *W* into four clusters, namely, the normal skin zone, the skin disease zone, and the zone that is mistakenly cut. Lastly, take each one in the four clustering forms as a large unit, among which each contains a smaller number of units. Then, look for one of the best smaller unit in the four larger units and take it as cluster center; continue to update the cluster center until the last center is no longer changed. The results are shown in Figure 11.

**Figure 12:**
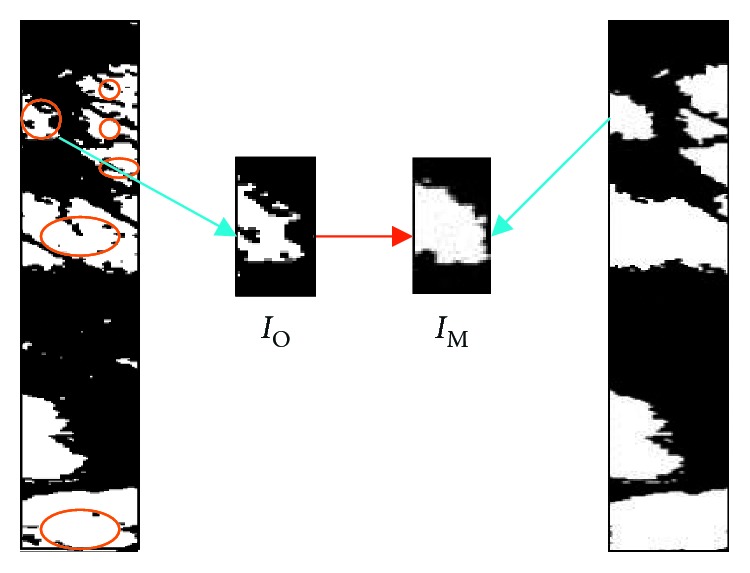
Morphological operation.

**Figure 13:**
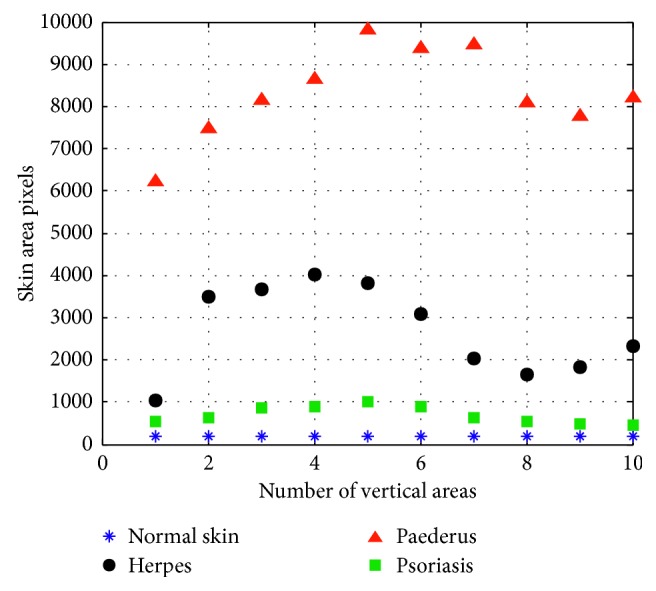
The calculation result of three skin diseases.

Figure 11 shows the result of segmentation. Marker-controlled watershed segmentation that is fusion-based can divide the lesion of skin disease in an effective way. Considering there are still some black flecks which will have a certain impact on the accuracy of experiments, in this paper, morphological approach is utilized to process images, thus making the segmented image smoother so that it will be easier to count the black and white pixel in the certain area. In Figure 12, *I*_O_ and *I*_M_ of segmented area are picked up for inspecting after morphology processing. The variation of different skin disease can be recognized by means of calculating the pixel area, as it is shown in Figure 13.

In Figure 13, according to the statistics of these three kinds of skin diseases, it is shown that the pixel area of normal skin is nearly a straight line. Besides the pixel area of normal skin, the pixel area of psoriasis is of the greatest, herpes the second, and with that of paederus dermatitis being the lowest. These three diseases can be accurately identified through clear and distinct variation. However, if some herpes once gets severe, the area of injured skin could become larger. In this case, some certain errors may emerge if we solely adhere to the statistics of pixel area. Therefore, only by combining the features of texture with pixel area can the recognition rate of skin disease be improved.

### 2.4. Classification Based on SVM

SVM is the statistical method based on the statistical learning theory, which is suitable for the classification of small sample numbers. It can obtain the minimized training error and a confidence interval term by analyzing the given training set to predict the test set. In this paper, we use SVM to identify three different skin diseases. Firstly, the sample number and training number are selected from the extracted features (such as color feature, and texture feature), and then by using the rational kernel function of support vector machine, the classification model can be established.

In this paper, three common skin diseases herpes, dermatitis, and psoriasis are represented as Class I, Class II, and Class III, respectively. Texture feature classifier is the SVM1, lesion area feature classifier refers to SVM2, and integrated classifier is represented as SVM. On this foundation, we change the different penalty factors to classify the maize diseases. As a result, the optimal recognition effect can be realized when the penalty factor *C*=50. The optimal classification function and the kernel function are as follows:

(10) $$\begin{array}{c} f\left(x\right)=\mathrm{sgn}\left\{\left(\overset{n}{\underset{j=1}{\sum }}a_{j}y_{j}k\left(x_{i},\;\;x_{j}\right)\right)+b^{*}\right\},\\ k\left(x_{i},\;\;x_{j}\right)=\exp\left(-\frac{{\left\|x_{i}-x_{j}\right\|}^{2}}{2\sigma^{2}}\right), \end{array}$$

where *a*_*j*_ is a Lagrange multiplier, *b*^*∗*^ is the bias, and *k*(*x*_*i*_, *x*_*j*_) is a kernel function; *x*_*i*_ refers to the eigenvector that is obtained from the characteristic model; *y*_*j*_ refers to the results; and *σ* is a parameter value in the radial basis function.

## 3. Experiments and Analysis

To identify the classification, in this experiment, common skin diseases (herpes, dermatitis, and psoriasis) are selected as the research objects. Ninety images are also selected to be identified accordingly, including herpes, dermatitis, and psoriasis, thirty cases in each, together with twenty test samples and ten standard samples. In this paper, the combination of the color feature and the texture feature is employed to conduct the experiment, the results of which are compared with those in [21, 22]. The comparison is presented in Table 2.

Table 1 presents the results of different recognition algorithms. It is used to identify images on twenty test samples and ten standard samples of each skin disease. In the study by Guo and Huo [22], the recognition accuracy is relatively low, with that of three diseases being at 75%, 80%, and 80%. De et al. [21] mainly applies the method of unidirectional texture feature recognition, which can cause the relatively large errors. While in the study by De et al. [21], the method of support vector machine and genetic algorithm, with a rather ideal efficiency, are utilized for recognition, with the recognition accuracy rate being 85%, 80%, and 85%. In this paper, the color feature and the texture feature can be used to make up for the weakness of the unidirectional recognition so that the recognition rate can reach to 90% and more, which greatly improves the accuracy. Ten local vertical images after segmentation can be obtained by the vertical image segmentation, therefore improving the accuracy of skin diseases identification.

## 4. Conclusions

In this paper, the analysis method of vertical image segmentation is employed to identify three common skin diseases. A number of irrelevant variables can be reduced through image filtering, image rotation, and Euclidean distance transformation applied in image preprocessing. Then, the perpendicular line for each point on the main axis can be determined. And, the epithelium can be divided into ten vertical image regions. Based on this, the grey-level co-occurrence matrix is adopted to extract the texture feature, and the area pixel method is applied to extract the characteristics of the lesion area. Finally, the support vector machine is utilized to classify the data of three different skin diseases according to the features of the texture and the lesion area, achieving a more ideal accuracy of recognition. Nevertheless, the paper concentrating on herpes, dermatitis, and psoriasis does not consider the different symptoms caused by the same kind of skin disease. For instance, eczema, herpes, and rubella all belong to the same series. Therefore, it will be the focus of next step to recognize different types of skin diseases of the same kind of series by using image processing technique.

## Figures and Tables

**Table 1:** Texture features of different skin disease.

	Contrast		Correlation		Entropy		Uniformity		Energy	
	Minimum	Maximum	Minimum	Maximum	Minimum	Maximum	Minimum	Maximum	Minimum	Maximum
Normal skin	0.2053	0.3805	3.589	3.8917	1.0696	1.8224	3.8105	3.8974	843.7668	1437.6
Herpes	41.7036	178.2016	3.5308	3.7852	0.1355	0.5163	2.9878	3.6857	93.0058	345.2991
Paederus	51.676	87.3321	2.8167	3.2025	0.0741	0.1691	2.928	3.125	53.2181	121.5074
Psoriasis	8.713	40.3506	1.8612	3.6789	0.1849	0.5666	3.0617	3.4794	36.9206	205.6441

**Table 2:** The different methods to identify the results.

Method	Herpes			Dermatitis			Psoriasis		
	Number of test	Recognition numbers	Recognition rate (%)	Number of test	Recognition numbers	Recognition rate (%)	Number of test	Recognition numbers	Recognition rate (%)
Reference [22]	20	15	75	20	16	80	20	16	80
Reference [21]	20	17	85	20	16	80	20	17	85
The proposed method	20	17	85	20	18	90	20	19	95

## Data Availability

The data used to support the findings of this study are available from the corresponding author upon request.
